# A Correlational Study of Scoliosis and Trunk Balance in Adult Patients with Mandibular Deviation

**DOI:** 10.1371/journal.pone.0059929

**Published:** 2013-03-29

**Authors:** Shuncheng Zhou, Juanjuan Yan, Hu Da, Yang Yang, Na Wang, Wenyong Wang, Yin Ding, Shiyao Sun

**Affiliations:** 1 Department of Orthodontics, The 161th Hospital of PLA, Wuhan, China; 2 Department of Orthodontics, School of Stomatology, The Fourth Military Medical University, Xi’an, China; 3 Department of Prosthetics, School of Stomatology, The Fourth Military Medical University, Xi’an, China; 4 Department of Orthopedics, The 82nd Hospital of PLA, Huaian, China; 5 Department of Cardiovascular Surgery, Xijing Hospital, The Fourth Military Medical University, Xi’an, China; 6 Department of Physiology and Pathophysiology, The Fourth Military Medical University, Xi’an, China; The University of Tennessee Health Science Center, United States of America

## Abstract

Previous studies have confirmed that patients with mandibular deviation often have abnormal morphology of their cervical vertebrae. However, the relationship between mandibular deviation, scoliosis, and trunk balance has not been studied. Currently, mandibular deviation is usually treated as a single pathology, which leads to poor clinical efficiency. We investigated the relationship of spine coronal morphology and trunk balance in adult patients with mandibular deviation, and compared the finding to those in healthy volunteers. 35 adult patients with skeletal mandibular deviation and 10 healthy volunteers underwent anterior X-ray films of the head and posteroanterior X-ray films of the spine. Landmarks and lines were drawn and measured on these films. The axis distance method was used to measure the degree of scoliosis and the balance angle method was used to measure trunk balance. The relationship of mandibular deviation, spine coronal morphology and trunk balance was evaluated with the Pearson correlation method. The spine coronal morphology of patients with mandibular deviation demonstrated an “S” type curve, while a straight line parallel with the gravity line was found in the control group (significant difference, *p<0.01*). The trunk balance of patients with mandibular deviation was disturbed (imbalance angle >1°), while the control group had a normal trunk balance (imbalance angle <1°). There was a significant difference between the two groups (*p<0.01*). The degree of scoliosis and shoulder imbalance correlated with the degree of mandibular deviation, and presented a linear trend. The direction of mandibular deviation was the same as that of the lateral bending of thoracolumbar vertebrae, which was opposite to the direction of lateral bending of cervical vertebrae. Our study shows the degree of mandibular deviation has a high correlation with the degree of scoliosis and trunk imbalance, all the three deformities should be clinically evaluated in the management of mandibular deviation.

## Introduction

Patients with a mandibular deviation with a lateral shift in the midline of the mandible may show asymmetric temporomandibular joint (TMJ) conditions because the shape of the face and occlusion are not symmetrical [1]–[3]. Mandibular deviation can not only lead to TMJ disorders, facial asymmetry, and unbalanced craniofacial muscle strength, but also affect the balance and coordination of symmetric trunk muscles. This can lead to asymmetries of the head, neck, shoulder, and waist when sitting or walking [4]–[6]. Mandibular deviation also affects oral function and body appearance [7]. Previous studies have shown that patients with mandibular deviation often have abnormal morphology of their cervical vertebrae [8], [9]. However, the relationship of mandibular deviation, scoliosis, and trunk balance has not been studied. Therefore, mandibular deviation is usually treated as the sole pathology, which leads to poor clinical efficiency [10].

According to previous studies, the scoliotic curves in the frontal plane - through the head posture tilted sideward - contribute to the development of the different dentofacial asymmetries [11], [12]. Ito G and colleague [13] have reported that body posture is closely associated with the function of head-supporting system. The cervical spine and muscles serve to stabilize head posture and play an important role in the complex and diverse movements of the head. The morphologic changes resulted in dysfunction of the atlanto-occipital and atlantoaxial joints, limited head rotation, and asymmetric masticatory muscle activity and condylar movement, chronically causing asymmetric of the condyle and other joint structures [14], [15]. The resultant disharmony of jaw movement may lead to the development of recurrence of mandibular deviation. We have also come across many recurrence cases of mandibular deviation after treatment. These cases indicated that the scoliosis and trunk imbalance may account for the recurrence of mandibular deviation.

In this study, we investigated the relationship of spine coronal morphology and trunk balance in adult patients with mandibular deviation, and compared them to healthy volunteers.

## Materials and Methods

### Patients

**Figure 1:**
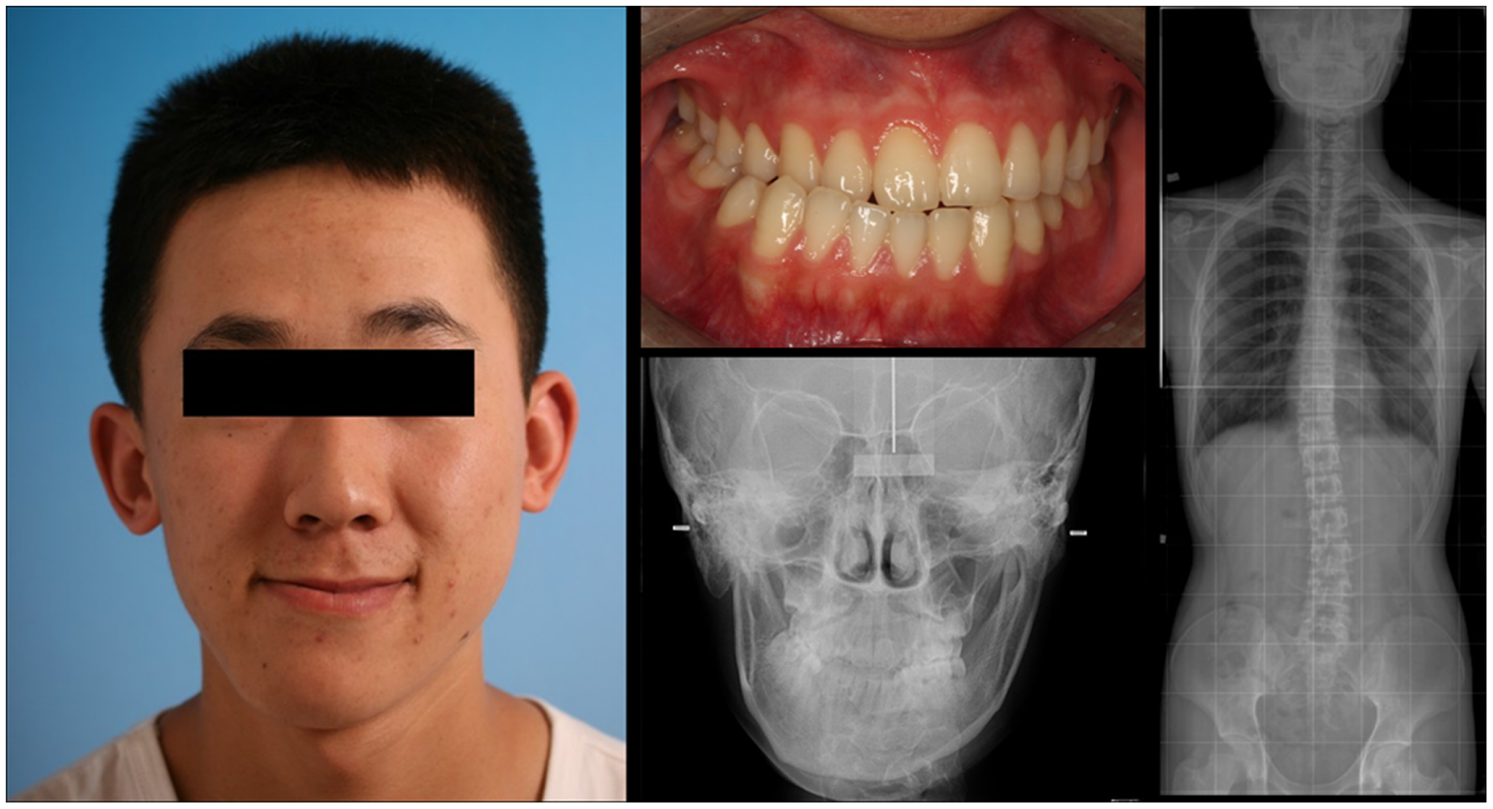
Image from a typical patient with mandibular deviation. Anterior X-ray film. The lengths of the mandibular rami have an obvious difference. The vertical distance from the chin ridge to the inferior orbital fissure line is greater than 2 mm.

This study was conducted according to the Declaration of Helsinki principles and approved by the Joint Committee on Clinical Investigation of our Stomatological College. Written informed consent was received from patients or their guardians before inclusion in the study. From October 2009 to October 2010, 35 adult patients only with skeletal mandibular deviation who visit our out-patient clinic attended our department were included in this study. These patients (19 males and 16 females) were from 18 to 30 (mean: 23.5±4.8) years old. Diagnostic criteria of mandibular deviation were observed from anterior X-ray films, and were defined as the demonstration of an obvious difference in the lengths of the two mandibular rami, and a vertical distance from the chin ridge to inferior orbital fissure (IOF) line greater than 2 mm. There were no special occlusive requirements in these patients (Fig. 1).

**Figure 2:**
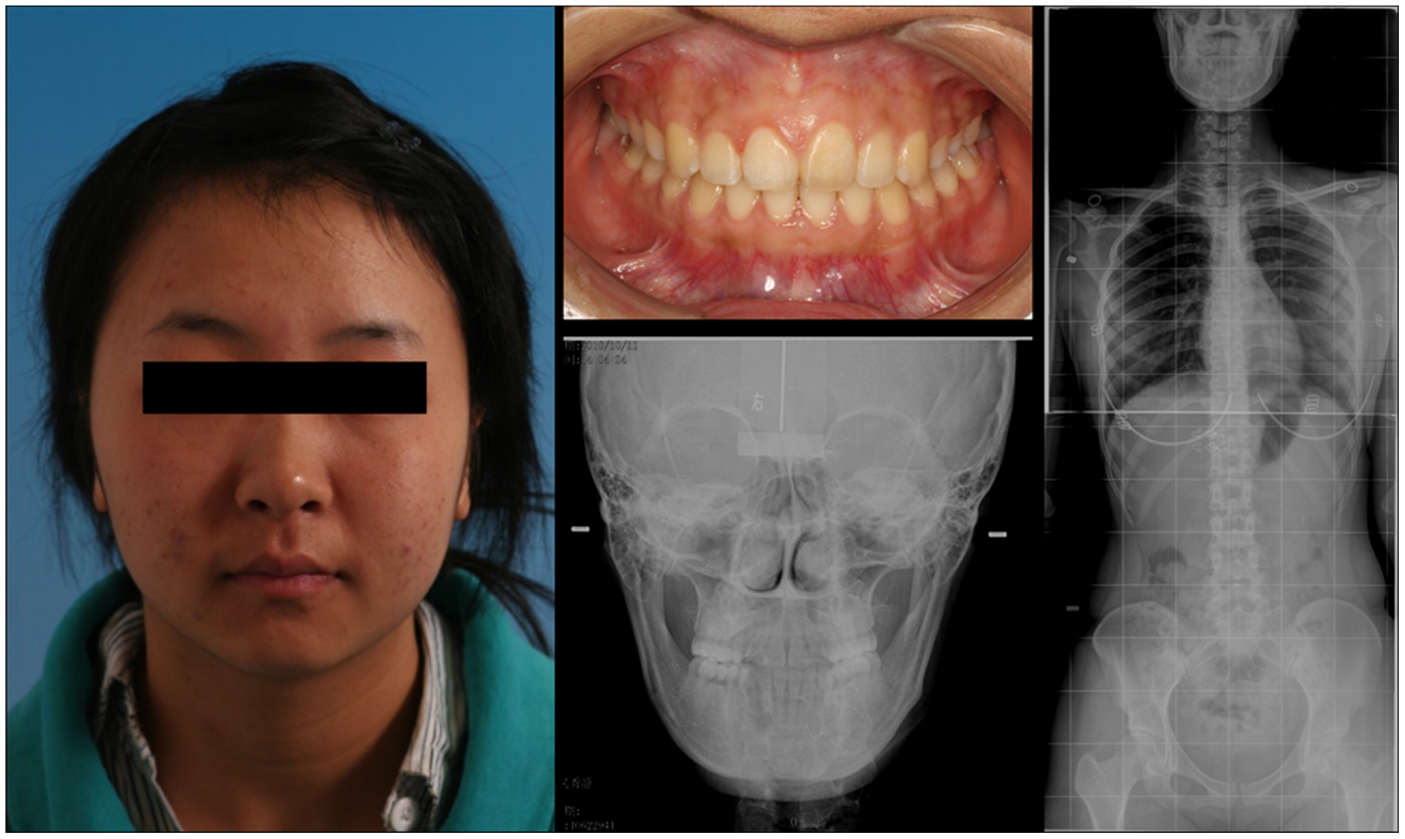
Image from a typical volunteer.

The control group consisted of 10 volunteers were selected from the Master’s graduate program at our university. Volunteers selected had no orthodontic history, correct facial appearance and no facial deformity, neat teeth rows, the same midline up and down dentition, neutral occlusion, and a normal relationship of lamination and covering. Volunteers were 21 to 28 (mean: 25±3.6) years old (Fig. 2).

Evaluation consisted of anterior X-ray films of the head (maintaining central occlusion and head posture) and posteroanterior X-ray films of the spine. These studies were taken by two technicians, respectively.

### X-ray Films Technique

Anterior X-ray films of the head. Both ear canals were fixed with earplugs, and the orbitomeatal line was oriented parallel to the ground. The centerline of the radiologic study was parallel to the ground. Radiologic studies were performed using the Philips 8K70 with an exposure distance of 36 cm and exposure conditions of 63 kV and 100 mA.

Posterioranterior X-ray films of the spine. Positioning consisted of standing closely back against the image plates, arms hanging naturally, palms forward, and head looking straight ahead. The vertical line of the X-ray was arranged parallel to the body’s vertical line. Radiologic studies were performed using the Philips Digital Diagnost VS, with an exposure distance of 200 cm, and exposure conditions of 66 kV and 200 mA.

### Landmarks and Lines in the X-ray Films

**Figure 3:**
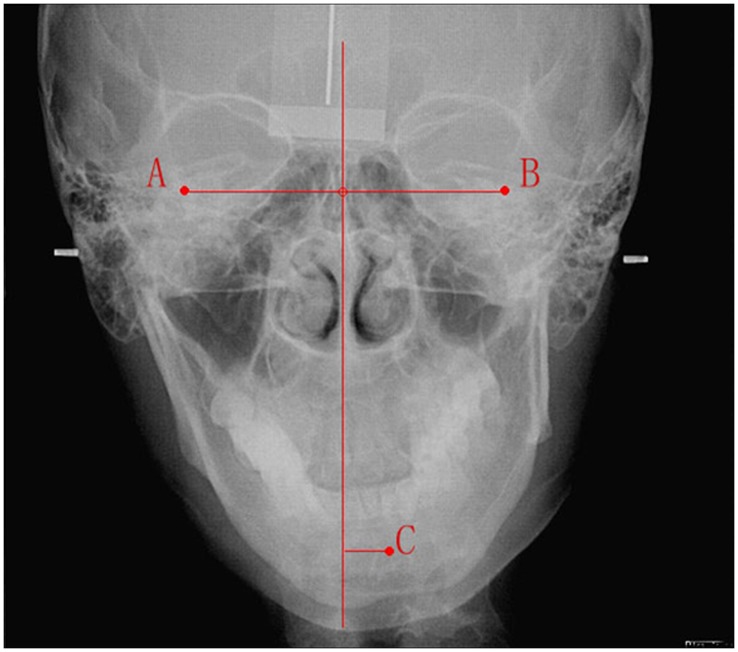
Anterior X-ray film of head. The midpoints of the inferior orbital fissures were designated as points A and B, and the midpoint of the chin ridge was as point C.

**Figure 4:**
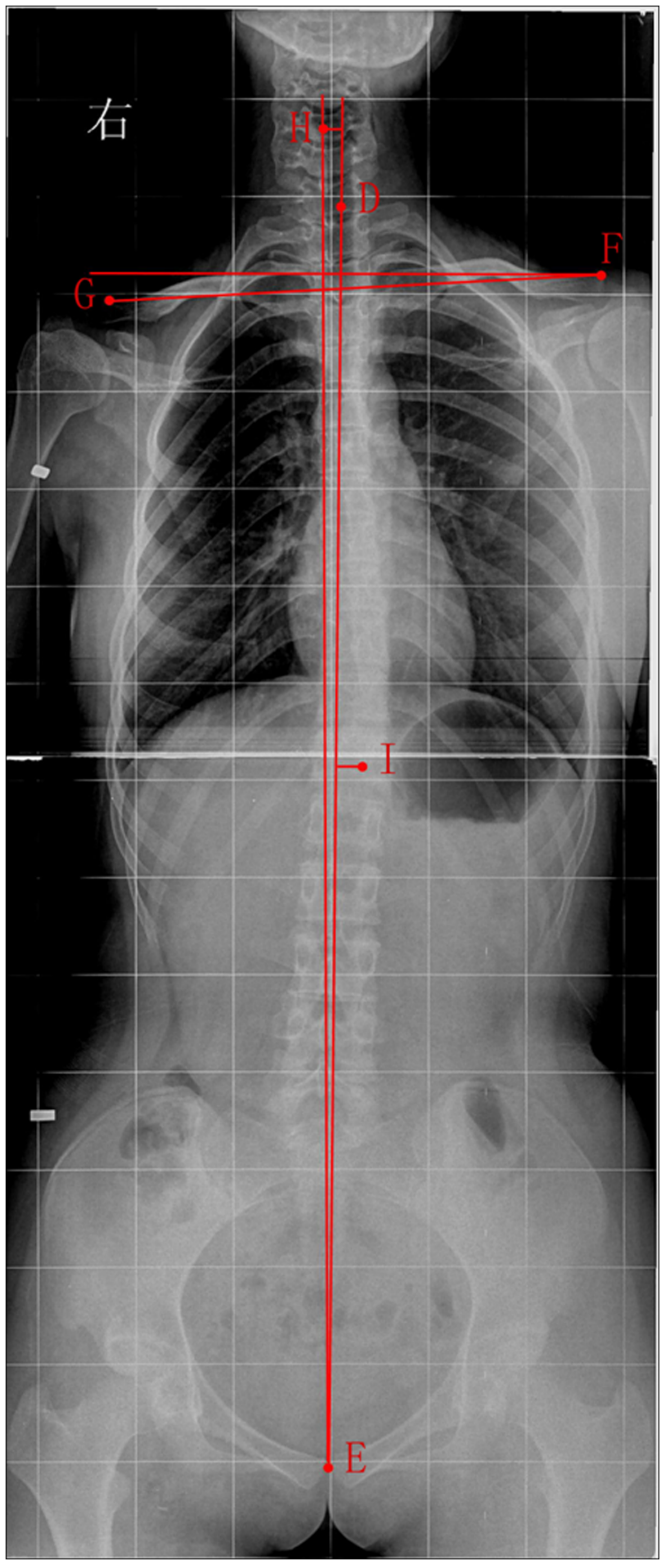
Standing anteroposterior X-ray film of the full-length spine. The midpoint of the 7th cervical vertebra was designated as point D, the midpoint of pubic symphysis point E, and the shoulder peaks as points F and G. In the cervical and thoracic vertebrae, the midpoints of the upper most scoliotic vertebra were designated as points H and I.

Patient films were measured using Digimizer image measurement software. In the anterior X-ray film of head, the midpoint of each intraocular fissure was designated as points A and B, and the midpoint of the chin ridge as point C (Fig. 3). In the standing anteroposterior study of the full-length spine, the vertical midpoint of the 7th cervical vertebra was designated as point D, the vertical midpoint of the pubic symphysis point E, and the two shoulder peaks points F and G. In the cervical and thoracic vertebrae, the horizontal midpoint of the uppermost or first scoliotic vertebra were designated as points H and I (Fig. 4). A midperpendicular line was drawn connecting points A and B. Points D and E were connected with the gravity line. A horizontal line was drawn through points F and G. There were a total of nine landmarks and six lines.

### Diagnostic Criteria

The degree of scoliosis was measured by the axis distance method [16] and the trunk balance was measured by the balance angle method [17].

Using the anterior X-ray film of the head, the vertical distance between point C and line AB (which reflects the degree of mandibular deviation) was determined. Using the standing anteroposterior X-ray film of the full-length spine, the vertical distance between point H or I and line DE, the vertex angle of line FG and the horizontal line (which can reflect an imbalanced angle in the shoulders), and the vertex angle of line DE and the gravity line (which can reflect an imbalanced angle of the trunk) were determined.

### Statistical Analysis

All of the values are presented as mean ± standard error of mean (SEM). The data in the male and female groups had no significant difference, and so were combined into one group. The data in the mandibular deviation group and control group were analyzed by statistical methods.

Student t-test and Pearson correlation methods were used to determine significance. A difference of P<0.05 was considered statistically significant.

## Results

### Results from Anterior X-ray Films of the Head

The vertical distance between point C and line AB in the control group was less than 2 mm (average distance: 0.53±0.10 mm) in all members. The vertical distance from point C to line AB in the mandibular deviation group was more than 2 mm in all members. The average distance was 7.45±0.46 mm (range: 2.94–13.32 mm). There was significant difference between the two groups (*P<0.01*). In the mandibular deviation group, 20 patients leaned to the right and 15 patients leaned to the left (Table 1).

**Table 1:** The difference analysis between Patients group and Control group.

	Group	Number	Mean	Standard deviation	Standard error	P value
The degree of mandibular deviation(mm)	Case	35	7.45	2.73	0.46	5.99e−10
	Control	10	0.53	0.32	0.10	
The scoliotic degree of cervical vertebra(mm)	Case	35	3.24	1.10	0.19	3.42e−09
	Control	10	0.61	0.33	0.10	
The scoliotic degree of thoracolumbar vertebra(mm)	Case	35	9.40	2.43	0.41	2.03e−12
	Control	10	1.76	0.81	0.25	
The imbalanced angle of shoulders(°)	Case	35	2.34	0.80	0.14	6.22e−10
	Control	10	0.30	0.17	0.05	
The imbalanced angle of trunk(°)	Case	35	1.51	0.30	0.05	6.28e−15
	Control	10	0.34	0.21	0.06	

### Results from the Standing Anteroposterior X-ray Films of the Full-length Spine

**Figure 5:**
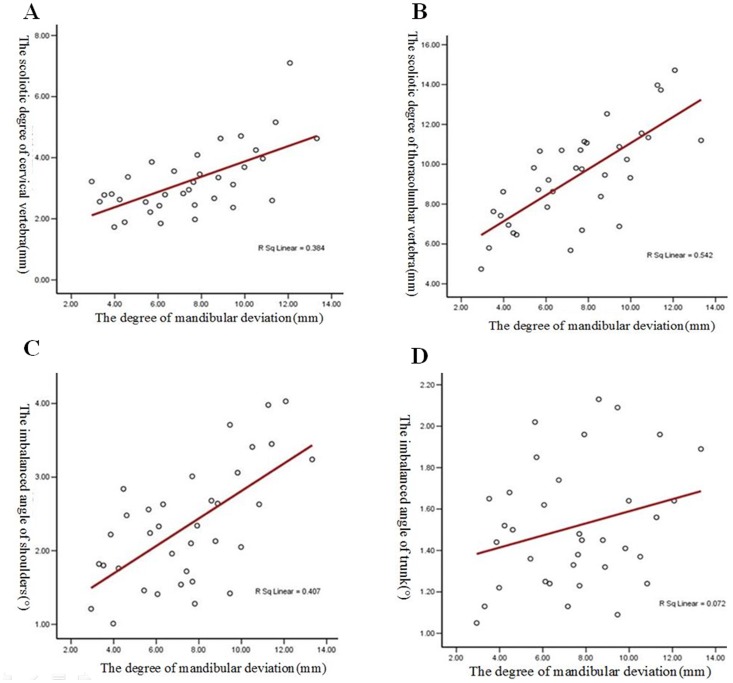
The correlation analysis of mandibular deviation with scoliosis or trunk balance. **A.** The degree of scoliosis in the cervical vertebrae had a positive, linear correlation with the degree of mandibular deviation. **B.** The degree of scoliosis in the thoracolumbar vertebrae had a positive, linear correlation with the degree of mandibular deviation. **C.** The imbalanced angle of the shoulders had a positive, linear correlation with the degree of mandibular deviation. **D.** The imbalanced angle of the trunk had a positive correlation with the degree of mandibular deviation, but there was no linear trend.

In the control group, the scoliotic distance in the cervical vertebrae varied from 0.32 to 1.10 mm (average: 0.61±0.10 mm). The scoliotic distance in the thoracolumbar vertebrae varied from 0.63 to 3.20 mm (average: 1.76±0.25 mm). In the mandibular deviation group, the scoliotic distance in the cervical vertebrae varied from 1.73 to 7.10 mm (average: 3.24±0.19 mm). The scoliotic distance in the thoracolumbar vertebrae varied from 5.68 to 14.72 mm (average: 9.40±0.41 mm). Above all, the spinal coronal morphology of patients with mandibular deviation demonstrated an “S” type curve. The control group had a straight line parallel to the gravity line. The difference was statistically different. The degree of scoliosis in the cervical and thoracolumbar vertebrae was statistically different between the control and patient groups (*P<0.01*, Table 1). In addition, the degree of scoliosis in the cervical and thoracolumbar vertebrae had a positive, linear relation with the degree of mandibular deviation (Figs. 5A and 5B and Table 2).

**Table 2:** The Bivariate Pearson correlation analysis.

	Analysis method	The scoliotic degree of cervical vertebra(mm)	The scoliotic degree of thoracolumbar vertebra(mm)	The imbalanced angle of shoulders(°)	The imbalanced angle of trunk(°)
The degree of (mm)	Pearson Correlation	0.62	0.74	0.64	0.27
	Sig.(2-tailed)	0.00**	0.00**	0.00**	0.12

** correlation is significant at the 0.01 level (2-tailed test).

In the control group, the imbalanced angle of the shoulders varied from 0.05 to 0.62° (average angle: 0.30±0.05°). The imbalanced angle of the trunk varied from 0.04 to 0.65° (average angle: 0.34±0.06°). In the mandibular deviation group, the imbalanced angle of shoulders varied from 1.01 to 4.03° (average angle: 2.34±0.14°). The imbalanced angle of the trunk varied from 0.15 to 2.13° (average angle: 1.51±0.05°), where 0°–1° is normal and greater than 1° means loss of balance [5]. The imbalanced angle of the shoulders and the imbalanced angle of the trunk was statistically different between the control and patient groups (*P<0.01*, Table 1). The imbalanced angle of the shoulders had a positive, linear correlation with the degree of mandibular deviation (Fig. 5C and Table 2). The imbalanced angle of the trunk had a positive correlation with the degree of mandibular deviation, but didn’t present a linear trend (Fig. 5D, Table 2).

In addition, the direction of scoliosis had a high degree of correlation with the direction of mandibular deviation. The direction of mandibular deviation was in the same direction as the lateral bending of thoracolumbar vertebrae, which was opposite to the direction of lateral bending of cervical vertebrae.

## Discussion

Mandibular deviation has been related to vertical asymmetries, such as asymmetry of the frontal maxillary plane angles [18]. Asymmetric functional loading of the temporomandibular joints is an important causative factor in TMJ disease [19], [20]. It has been reported that patients with mandibular deviation also have functional and morphologic asymmetries [21], [22]. Mandibular deviation is strongly related to the condylar path, which is steeper on the nondeviated side during protrusive excursion and maximum opening and closing movements [23]. Mandibular position is believed to be involved in anti-gravity muscle activity and posture control via various neurophysiologic and anatomic mechanisms [24].

Horizontal deviation in mandibular position interfered with stability of upright posture on an unstable platform, suggesting that changes in the stomatognathic system affects dynamic balance [24]. The abnormal head position can affect the mandible position, which can then affect the posture of the head, neck, and trunk [25]. Studies have indicated that the tilt rates of the occlusal plane, mandibular plane, and the Frankfort plane have a significant relationship with the tilt rates of the cervical vertebrae tangent and odontoid process tangent [26]. Ozbek, et al. confirmed the relationship between posture and structure of the head is controlled by functional factors related to “forward cervical posture” and “vertical cervical posture” [27]. Huggare also found that abnormal morphology of the cervical vertebrae was closely related to mandibular deviation [28]. Kondo has opined that preoperative functional assessments of mouth jaw muscles and neck muscles are essential in the treatment of patients with skeletal mandibular deviation, and that body posture has a relationship with the balance of muscles [9].

Mandibular deviation can be caused by teeth deformity, functional disability, and skeletal deformity [9]. Additionally, during childhood growth, facial asymmetry will gradually change and be stable when the patient reaches adulthood [29]. In order to improve the accuracy of our study, only patients with skeletal mandibular deviation were enrolled in this study. Therefore, adult patients were selected as objects in this study. Actually, 321 adult patients with skeletal mandibular deviation at our center were excluded from this study, they had no scoliosis or trunk imbalance. According to our survey, about 9.8% of the patients with skeletal mandibular deviation had scoliosis/trunk imbalance. While Lonstein JE and colleagues found that only 1.2% of one-quarter of a million children in Minnesota were detected to have scoliosis/trunk balance [30]. The difference between our and Lonsterin JE’s results was statistically different. These results suggest that there is a positive correlation between mandibular deviation and scoliosis/trunk balance.

Different techniques can be used to measure the degree of mandibular deviation. Identifying the typical landmarks in X-ray films is a commonly used method. The submental vertex radiograph method is a common measurement technique, however, the foramen spinosum cannot be located accurately by this method [31], [32]. Anterior X-ray films of the head and standing anteroposterior X-ray films of the full-length spine were used in our study. These methods target the landmarks of the body more accurately, increasing the reliability of the results. In this study, 35 adult patients with skeletal mandibular deviation and 10 healthy volunteers were evaluated with anterior X-ray films of the head and posteroanterior X-ray films of the spine. The axis distance method was used to measure the degree of scoliosis and the balance angle method was used to measure the trunk balance. The relationship between mandibular deviation, spine coronal morphology, and trunk balance was examined.

The spinal coronal morphology of patients with mandibular deviation demonstrated an “S” type curve. The control group had a straight line parallel to the gravity line. The difference was statistically different. The degree of mandibular deviation and the degree of scoliosis had a linear correlation, suggesting that mandibular deviation may lead to morphological changes of the spinal coronal plane. Therefore, in the process of treating such patients, orthodontic physicians should not only examine mandibular deviation, but also evaluate morphological changes in the spinal coronal plane. If the patient has a serious deformity, correction of spinal shape is indicated.

The trunk balance of patients with mandibular deviation was disturbed (imbalance angle >1°), while that of the control group was not (imbalance angle <1°). There was a statistically significant difference between the two groups. Additionally, the imbalanced angle of the shoulders had a positive, linear correlation with the degree of mandibular deviation. The imbalanced angle of the trunk had a positive correlation with the degree of mandibular deviation, but did not present a linear trend. These results suggest that mandibular deviation affects the trunk balance of the body. The more severe the degree of mandibular deviation, the greater impact on trunk balance. Therefore, in the clinical treatment of mandibular deviation, orthodontic physicians should not only correct mandibular deviation, but also correct abnormal postures of sitting, standing, and walking.

We also found that the direction of mandibular deviation was the same as that of the lateral bending of the thoracolumbar vertebrae, which was opposite to the direction of lateral bending of cervical vertebrae. These results suggest mandibular deviation has an adverse impact on the coronal plane shape of spine and trunk balance. In order to prevent the progression of mandibular deviation to bony asymmetry, it should be diagnosed and treated early. If mandibular deviation has progressed to bony asymmetry, the patient should be treated with a combination therapy of orthodontic - orthognathic treatment as soon as possible.

### Conclusions

In summary, we find that all patients with skeletal mandibular deviation in our study have varying degrees of scoliosis and trunk imbalance. Most importantly, the degree of mandibular deviation has a high correlation with the degree of scoliosis and trunk imbalance. These results suggest the best patient management evaluates all three deformities. There are some limitations of our study. We cannot identify a cause and effect relationship between mandibular deviation, scoliosis, and trunk imbalance from this associative study. Nor can we identify which factor plays a leading role in the development of the disease. We hope to establish an appropriate animal model to explore the relationship of these factors, which can provide a more scientific basis for clinical treatment.
